# Crystal structure of [5-*n*-butyl-10-(2,5-di­meth­oxy­phen­yl)-2,3,7,8,13,12,17,18-octa­ethyl­porphyrin­ato]nickel(II)

**DOI:** 10.1107/S2056989015020058

**Published:** 2015-10-31

**Authors:** Keith J. Flanagan, Ebrahim M. Mothi, Lisa Kötzner, Mathias O. Senge

**Affiliations:** a School of Chemistry, SFI Tetrapyrrole Laboratory, Trinity Biomedical Sciences Institute, 152-160 Pearse Street, Trinity College Dublin, The University of Dublin, Dublin 2, Ireland; b Center for Scientific and Applied Research, PNS College of Engineering and Technology, Melathediyoor, Tirunelveli 627 152, India

**Keywords:** crystal structure, 2,3,7,8,12,13,17,18-octa­ethyl­porphyrin, 5,10-disubstituted porphyrins, Ni^II^ porphyrin, normal structural decomposition (NSD) method, nickel(II) complexes

## Abstract

The asymmetric unit of the title nickel(II) porphyrin, [Ni(C_48_H_60_N_4_O_2_)], contains one independent mol­ecule. The average Ni—N bond length is 1.917 (13) Å. The mol­ecules are arranged in a closely spaced lattice structure in which neighbouring porphyrins are oriented in inversion-related dimers. The nickel(II) porphyrin is characterized by a significant degree of a *ruf*fled (*B*_1*u*_) conformation with small contributions from *sad*dle (*B*_2*u*_) and *wave* (*y*) [*E_g_*(*y*)], as determined using normal structural decomposition. Disorder in the 2,5-di­­meth­oxy­phenyl substituent was modelled over two positions with a 60% occupancy for the major moiety. One of the ethyl groups is also disordered over two positions and was modelled with the major moiety being present in 51.3% occupancy.

## Abstract 2

The title compound contains one independent mol­ecule which exhibits an overall *ruf*fled conformation, with an average Ni—N bond length of 1.917 (13) Å. The mol­ecules form a closely spaced lattice structure in which neighbouring porphyrins are oriented in inversion-related dimers.

## Chemical context   

The structural chemistry of porphyrin metal complexes is one of the largest explored areas of coordination chemistry. There are many studies available on metal coordination (Scheidt, 2008 ▸), aspects of macrocycle modification (Chmielewski & Latos-Grazynski, 2005 ▸), supra­molecular chemistry (Beletskaya *et al.*, 2009 ▸) and nonplanar systems (Senge, 2006 ▸). Highly substituted porphyrins (octa-, nano-, deca-, undeca- and dodeca­substitued porphyrins) are of specific inter­est due to the increased nonplanarity which results in the alteration of photophysical properties due to distortions within the macrocyclic ring. Non-planar porphyrins have significantly lower fluorescence yields, larger Stokes shifts and a shorter lifetime of the lowest excited state than planar ones (Röder *et al.*, 2010 ▸). This has resulted in the synthesis and structure of numerous highly substituted porphyrins for biomimetic studies (Senge, 2006 ▸; Senge *et al.*, 2015 ▸).
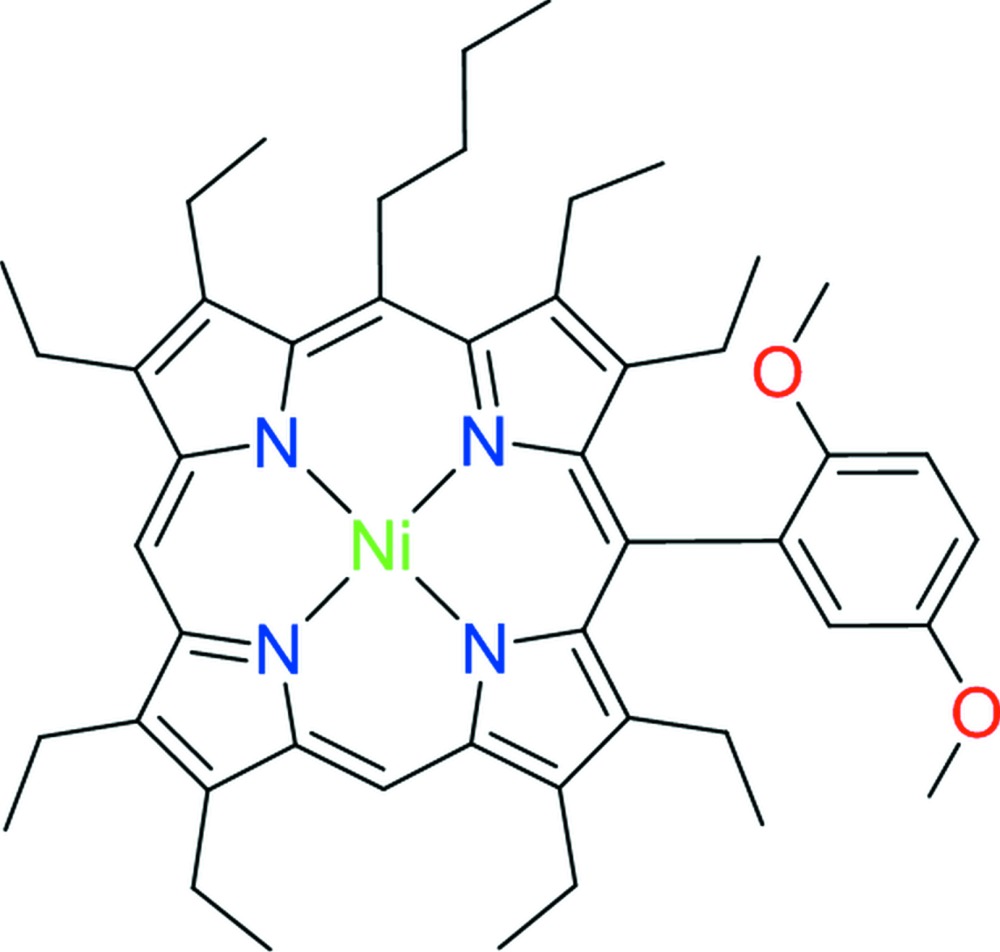


## Structural commentary   

**Figure 1:**
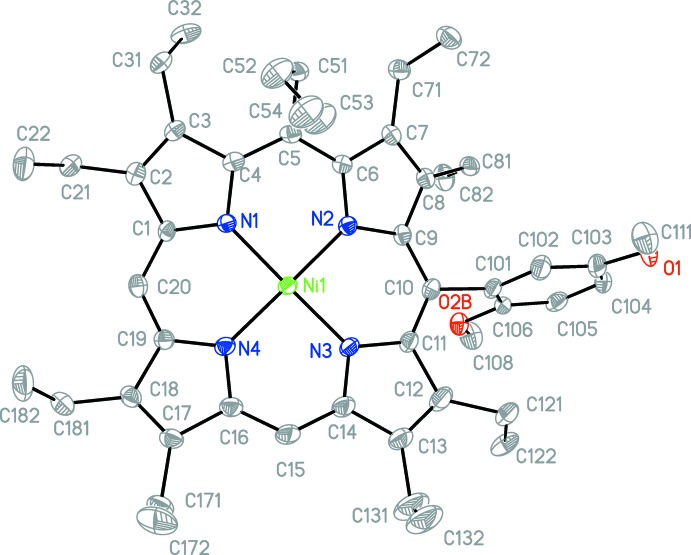
The mol­ecular structure of the title compound (only the major parts of the disordered substituents are shown). Displacement ellipsoids are drawn at the 50% probability level.

The title compound contains one mol­ecule in the asymmetric unit. The β-ethyl groups are either orientated above or below the plane. Ethyl groups on pyrrole rings next to a substituted *meso*-position alternate, whereas ethyl residues neighbouring an unsubstituted *meso*-position are orientated in the same direction (Fig. 1 ▸).

The average Ni—N distance is 1.917 (13) Å. The largest deviation occurs at the Ni—N2 bond [1.906 (2) Å], which lies between both substituted *meso*-positions. These lengths are comparable to those in other similar nickel porphyrins, such as [2,3,7,8,12,13,17,18-octa­ethyl-5-(tri­fluoro­meth­yl)porphyrinato]nickel(II), which has an average Ni—N bond length of 1.925 Å (Suzuki *et al.*, 2014 ▸). The angles between the α carbons (C_α_) and the *meso* carbon (C_*m*_) can be used to determine structural differences between similar porphyrins and differences within the individual porphyin structure. The C_α_—C_*m*_(but­yl)—C_α_ angle of 119.12 (2)° is smaller than the C_α_—C_*m*_(H)—C_α_ angle, and the C_α_—C_*m*_(2,5-di­meth­oxy­phen­yl)—C_α_ angle at 123.2 (2)° is similar to both C_α_—C_*m*_(H)—C_α_ angles, 122.1 (3)° (C20) and 124.8 (3)° (C15). The 2,5-di­meth­oxy­phenyl group is tilted at an angle of 75.80 (7)° from the 24-atom least-squares plane of the porphyrin ring.

**Figure 2:**
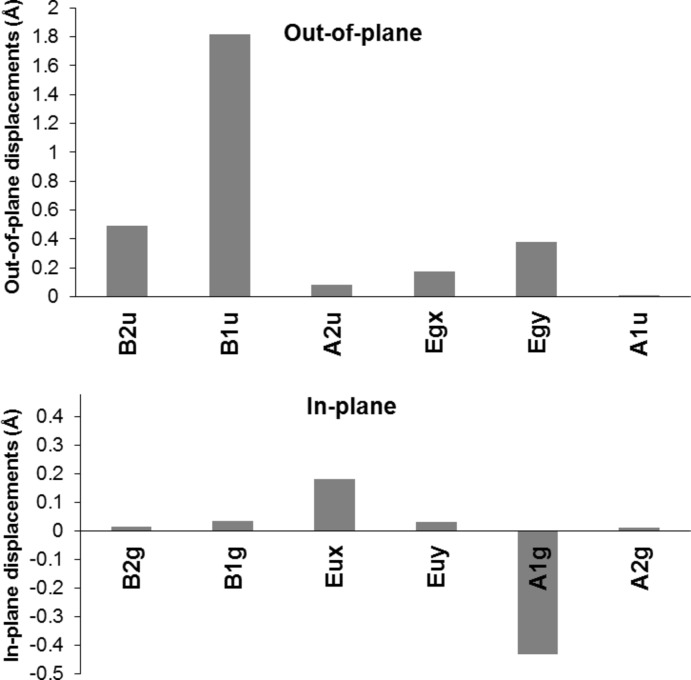
Normal structural decomposition (NSD) analysis of the title compound.

A conformational analysis was performed using the NSD (normal structural decomposition) method developed by Shelnutt and co-workers (Shelnutt *et al.*, 1998 ▸). The conformation is characterized by a significant degree of *ruf*fled (*B*_1*u*_) with small contributions from *sad*dle (*B*_2*u*_) and *wave* (*y*) [*E_g_*(*y*)] (Fig. 2 ▸). There are also minor contributions from *wave* (*x*), [*E_g_*(*x*)] and *dom*ed (*A*_2*u*_), which is similar to both highly substituted and other Ni(II) porphyrins (Senge *et al.*, 1992 ▸, 2000 ▸; Senge & Bischoff, 2001 ▸). Contributions are also evident in the *A*_1*g*_ in-plane distortion with smaller contributions from the *E_u_*(x). The tilt of the pyrrole rings against the 24-atom plane are N1 [24.85 (8)°], N2 [25.22 (8)°], N3 [15.79 (10)°] and N4 [17.58 (8)°], with the highest deviation from the mean plane associated with the pyrrole rings closest to the butyl group at C5. The maximum deviations from the least-squares plane are associated with the *meso* C atoms. C5 deviates from the least-squares plane by 0.880 (2) Å, whereas C10, C15 and C20 deviate from the plane at 0.551 (2), 0.512 (3) and 0.667 (2) Å, respectively. Table 1 ▸ shows the deviation of all atoms in the 24-atom ring.

## Supra­molecular features   

**Figure 3:**
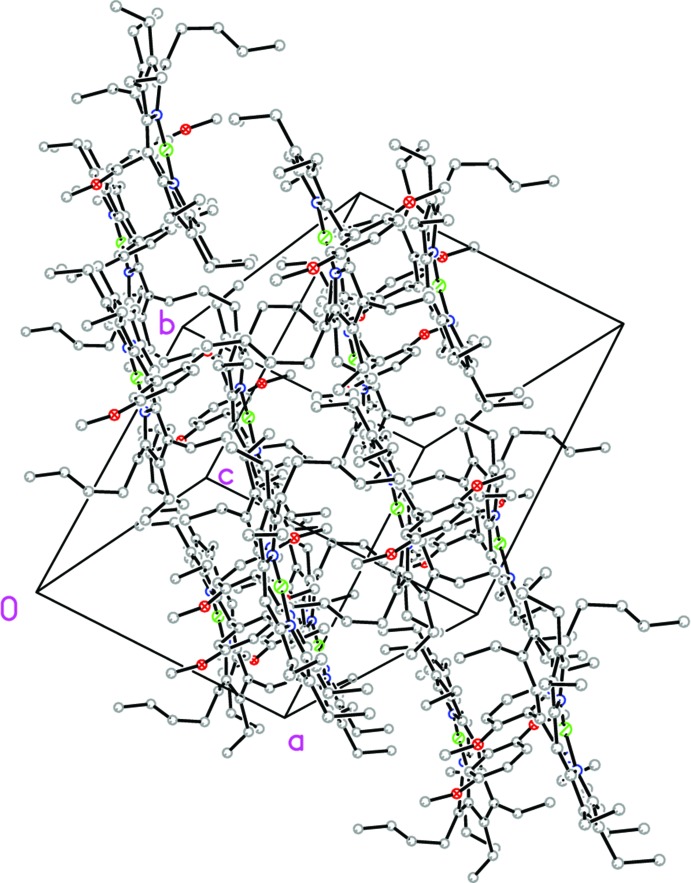
Crystal packing diagram of the title compound, showing the arrangement of inversion-related mol­ecules.

The unit cell of the title compound consists of two mol­ecules, each at a distance of 4.949 Å from the 24-atom mean plane of the other. The mol­ecules are arranged in a closely spaced lattice structure in which ethyl groups and butyl groups point towards each other to form a cage-like inversion-related dimer (Fig. 3 ▸). Mol­ecules are orientated in a head-to-tail fashion with an Ni⋯Ni separation of 8.9207 (8) Å. Short contacts between the H atoms of the meth­oxy groups and the N atoms (C111—H⋯N3) are present in the packing structure at a distance of 2.671 (3) Å. Other short contacts were found between the *n*-butyl group (C51 > C54) with the phenyl meth­oxy unit, specifically between H54*A*⋯C104, at 2.851 (4) Å, the meth­oxy group (O1 > C111) with the ethyl group (C181 > C182) between O1⋯H18*C* at 2.552 (4) Å, the meth­oxy group (O2*B* > C108) with the ethyl group (C21 > C22) between O2*B*⋯H22*A* at 2.486 (3) Å and the ethyl group (C121 > C122) with the C15 atom, between C15⋯H12*E* at 2.833 (3) Å. However, there are no π–π inter­actions or hydrogen bonds evident in the crystal structure.

## Database survey   

A search of the Cambridge Structural Database (CSD, Version 5.36, update November 2014; Groom & Allen, 2014 ▸) gave six hits for 5,10-disubstituted-2,3,7,8,12,13,17,18-octa­ethyl­porphyrins. Senge *et al.* (1992 ▸) reported the structure of [2,3,7,8,12,13,17,18-octa­ethyl-5,10-di(2-formyl­vin­yl)porph­yrin­ato]nickel(II), with an average Ni—N bond length of 1.900 Å and similar C_a_—C_m_(H)—C_a_ angles (122.85–123.58°) compared to the title compound. We also determined the structure of [5,10-di(*n*-but­yl)-2,3,7,8,12,13,17,18-octa­ethyl­porphyrinato]nickel(II) with and without deuterated chloro­form (Senge *et al.*, 2000 ▸). This compound exhibits an average Ni—N bond length of 1.900 Å and C_a_—C_m_—C_a_ angles similar to the title compound, 119.68–121.23° for substituted *meso*-positions and 122.58–122.65° for unsubstituted *meso*-positions. Related structures are those of 2,3,7,8,12,13,17,18-octa­ethyl-5,10-di­phenyl­porphyrin, (2,3,7,8,12,13,17,18-octa­ethyl-5,10-di­phenyl­porphyrinato)nickel(II) and (2,3,7,8,12,13,17,18-octa­ethyl-5,10-di­phenyl­porphyrinato)zinc(II) (Senge & Bischoff, 2001 ▸). The free base derivative shows larger C_α_—C_*m*_—C_α_ angles compared to the title compound. However, as expected, there is a noticeable difference in the angles involving substituted and unsubstituted *meso*-positions. The angles between substituted *meso*-positions are in the range 125–125.93°, and 126.90–127.48° for unsubstituted *meso*-positions. The Ni(II) derivative exhibits angles that are similar to the title compound, 122.12–122.35° for the substituted *meso*-positions and 123.42–123.78° for the unsubstituted *meso*-positions. The average Ni—N bond length of 1.923 Å is comparable to that of the title compound. The zinc derivative of this compound exhibits a larger average metal–nitro­gen bond length of 2.054 Å and wider C_α_—C_*m*_—C_α_ angles, 124.85–125.95° for the substituted *meso*-positions and 126.81–127.78° for unsubstituted *meso*-positions, as to be expected for zinc porphyrins.

Other highly substituted porphyrin structures include 5,15-disubstituted-2,3,7,8,12,13,17,18-octa­ethyl­porphyrins (Senge *et al.*, 2000 ▸; Kobayashi *et al.*, 1998 ▸; Jiang *et al.*, 1996 ▸; Zhu *et al.*, 1992 ▸) and 5,10,15-tris­ubstituted-2,3,7,8,12,13,17,18-octa­ethyl­porphyrins (Kalisch & Senge, 1998 ▸; Senge *et al.*, 2000 ▸; Senge & Bischoff, 2001 ▸).

## Synthesis and crystallization   

The title compound was prepared as reported previously (Senge *et al.*, 2000 ▸). 1-Bromo-2,5-di­meth­oxy­benzene (1 g, 4.6 mmol) was dissolved in tetra­hydro­furan (5 ml) and cooled to 193 K. The solution was treated dropwise with a solution of lithium in cyclo­hexane (2 *M*, 2.12 ml, 4.8 mmol). The solution was heated to room temperature and over the course of 1 h added to a solution of (5-butyl-2,3,7,8,12,13,17,18-octa­ethyl­porphyrinato)nickel(II) (100 mg, 0.14 mmol) yielding purple crystals of the title compound (60 mg, 0.08 mmol, 50%). The compound was recrystallized from a solution of 1%_vol_ MeOH in CH_2_Cl_2_ layered with hexane to yield single crystals suitable for X-ray diffraction.

## Refinement   

Crystal data, data collection and structure refinement details are summarized in Table 2 ▸. The C-bound H atoms were placed in their expected calculated positions and refined using a standard riding model: C—H = 0.95–0.98 Å, with *U*_iso_(H) = 1.5*U*_eq_(C) for methyl H atoms and 1.2*U*_eq_(C) for other H atoms. Disorder in the 2,5-di­meth­oxy­phenyl substituent was modelled over two positions with a 60% occupancy for the major moiety. The ethyl group at C12 was modelled over two positions with the major moiety being present in 51.3% occupancy. Restraints and constraints were used to model the disorder with *SHELXL2014* (Sheldrick, 2015*b* ▸) associated with the 2,5-di­meth­oxy­pheny group at C10 (EADP) and the ethyl group at C12 (SADI and EADP). The EADP command was also used to constrain the *n*-butyl chain at C5.

## Supplementary Material

Crystal structure: contains datablock(s) I, publication_text. DOI: 10.1107/S2056989015020058/wm5222sup1.cif

Structure factors: contains datablock(s) I. DOI: 10.1107/S2056989015020058/wm5222Isup2.hkl

CCDC reference: 1427139

Additional supporting information:  crystallographic information; 3D view; checkCIF report

## Figures and Tables

**Table 1:** Deviations of atoms from the least-squares plane of the porphyrin ring^*a*^

Atom	Deviation from the least-squares plane ()
C1	0.381(2)
C2	0.222(2)
C3	0.395(2)
C4	0.512(2)
C5	0.880(2)
C6	0.385(2)
C7	0.014(2)
C8	0.598(2)
C9	0.456(2)
C10	0.551(3)
C11	0.222(3)
C12	0.004(3)
C13	0.359(3)
C14	0.352(3)
C15	0.512(3)
C16	0.249(3)
C17	0.128(3)
C18	0.298(3)
C19	0.396(3)
C20	0.667(2)
N1	0.019(2)
N2	0.041(2)
N3	0.024(2)
N4	0.046(2)

Note: (*a*) Least-squares plane (*x*, *y*, *z* in crystal coordinates); 8.891(2)*x*+9.002(3)*y*+8.507(3)*z* = 10.726(2)

**Table 2:** Experimental details

Crystal data	
Chemical formula	[Ni(C_48_H_60_N_4_O_2_)]
*M*_r_	783.71
Crystal system, space group	Triclinic, *P* 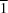
Temperature (K)	100
*a*, *b*, *c* ()	11.9496(6), 13.6692(6), 14.3909(7)
, , ()	72.018(2), 69.051(2), 89.558(2)
*V* (^3^)	2074.03(17)
*Z*	2
Radiation type	Mo *K*
(mm^1^)	0.51
Crystal size (mm)	0.30 0.14 0.03

Data collection	
Diffractometer	Bruker SMART APEXII area detector
Absorption correction	Multi-scan (*SADABS*; Bruker, 2014 ▸)
*T*_min_, *T*_max_	0.704, 0.745
No. of measured, independent and observed [*I* > 2(*I*)] reflections	50235, 7609, 4733
*R*_int_	0.103
(sin /)_max_ (^1^)	0.603

Refinement	
*R*[*F* ^2^ > 2(*F* ^2^)], *wR*(*F* ^2^), *S*	0.044, 0.088, 0.92
No. of reflections	7609
No. of parameters	525
No. of restraints	1
H-atom treatment	H-atom parameters constrained
_max_, _min_ (e ^3^)	0.85, 0.73

Computer programs: *APEX2* and *SAINT* (Bruker, 2014 ▸), *SHELXT* (Sheldrick, 2015*a* ▸), *SHELXL2014* (Sheldrick, 2015*b* ▸), *XP* in *SHELXTL* (Sheldrick, 2008 ▸) and *publCIF* (Westrip, 2010 ▸).

## supplementary crystallographic information

### Crystal data

**Table:**

[Ni(C_48_H_60_N_4_O_2_)]	*Z* = 2
*M**_r_* = 783.71	*F*(000) = 840
Triclinic, *P*1	*D*_x_ = 1.255 Mg m^−^^3^
*a* = 11.9496 (6) Å	Mo *K*α radiation, λ = 0.71073 Å
*b* = 13.6692 (6) Å	Cell parameters from 5872 reflections
*c* = 14.3909 (7) Å	θ = 2.3–29.9°
α = 72.018 (2)°	µ = 0.51 mm^−^^1^
β = 69.051 (2)°	*T* = 100 K
γ = 89.558 (2)°	Plate, orange
*V* = 2074.03 (17) Å^3^	0.30 × 0.14 × 0.03 mm

### Data collection

**Table:**

Bruker SMART APEXII area-detector diffractometer	7609 independent reflections
Radiation source: sealed tube	4733 reflections with *I* > 2σ(*I*)
Detector resolution: 8.258 pixels mm^-1^	*R*_int_ = 0.103
φ and ω scans	θ_max_ = 25.4°, θ_min_ = 1.6°
Absorption correction: multi-scan (*SADABS*; Bruker, 2014)	*h* = −14→14
*T*_min_ = 0.704, *T*_max_ = 0.745	*k* = −16→16
50235 measured reflections	*l* = −17→17

### Refinement

**Table:**

Refinement on *F*^2^	1 restraint
Least-squares matrix: full	Hydrogen site location: inferred from neighbouring sites
*R*[*F*^2^ > 2σ(*F*^2^)] = 0.044	H-atom parameters constrained
*wR*(*F*^2^) = 0.088	*w* = 1/[σ^2^(*F*_o_^2^) + (0.0356*P*)^2^] where *P* = (*F*_o_^2^ + 2*F*_c_^2^)/3
*S* = 0.92	(Δ/σ)_max_ = 0.001
7609 reflections	Δρ_max_ = 0.85 e Å^−^^3^
525 parameters	Δρ_min_ = −0.73 e Å^−^^3^

### Special details

**Table:**

Geometry. All e.s.d.'s (except the e.s.d. in the dihedral angle between two l.s. planes) are estimated using the full covariance matrix. The cell e.s.d.'s are taken into account individually in the estimation of e.s.d.'s in distances, angles and torsion angles; correlations between e.s.d.'s in cell parameters are only used when they are defined by crystal symmetry. An approximate (isotropic) treatment of cell e.s.d.'s is used for estimating e.s.d.'s involving l.s. planes.

### Fractional atomic coordinates and isotropic or equivalent isotropic displacement parameters (Å^2^)

**Table:**

	*x*	*y*	*z*	*U*_iso_*/*U*_eq_	Occ. (<1)
C1	0.0992 (2)	0.5331 (2)	0.5481 (2)	0.0174 (6)	
C2	0.0103 (2)	0.5576 (2)	0.6339 (2)	0.0195 (7)	
C3	0.0058 (2)	0.6612 (2)	0.6015 (2)	0.0184 (6)	
C4	0.0856 (2)	0.7003 (2)	0.4905 (2)	0.0181 (6)	
C5	0.0947 (2)	0.7989 (2)	0.4198 (2)	0.0179 (6)	
C6	0.1288 (2)	0.8118 (2)	0.3124 (2)	0.0174 (6)	
C7	0.0798 (2)	0.8822 (2)	0.2422 (2)	0.0202 (7)	
C8	0.1231 (2)	0.8625 (2)	0.1491 (2)	0.0202 (6)	
C9	0.2073 (2)	0.7866 (2)	0.1581 (2)	0.0180 (6)	
C10	0.2973 (2)	0.7647 (2)	0.0761 (2)	0.0190 (6)	
C15	0.5064 (3)	0.5136 (2)	0.2482 (2)	0.0331 (8)	
H15A	0.5838	0.4900	0.2292	0.040*	
C16	0.4260 (3)	0.4679 (2)	0.3497 (2)	0.0242 (7)	
C17	0.4371 (3)	0.3727 (2)	0.4247 (2)	0.0244 (7)	
C18	0.3325 (3)	0.3492 (2)	0.5088 (2)	0.0236 (7)	
C19	0.2583 (2)	0.4315 (2)	0.4878 (2)	0.0199 (7)	
C20	0.1495 (2)	0.4414 (2)	0.5591 (2)	0.0212 (7)	
H20A	0.1075	0.3825	0.6183	0.025*	
C21	−0.0577 (3)	0.4800 (2)	0.7411 (2)	0.0246 (7)	
H21A	−0.0622	0.4103	0.7345	0.029*	
H21B	−0.1411	0.4975	0.7683	0.029*	
C22	0.0016 (3)	0.4773 (2)	0.8199 (2)	0.0398 (9)	
H22A	−0.0446	0.4247	0.8877	0.060*	
H22B	0.0029	0.5452	0.8293	0.060*	
H22C	0.0843	0.4601	0.7933	0.060*	
C31	−0.0812 (2)	0.7179 (2)	0.6658 (2)	0.0244 (7)	
H31A	−0.0395	0.7853	0.6546	0.029*	
H31B	−0.1072	0.6769	0.7415	0.029*	
C32	−0.1926 (3)	0.7367 (2)	0.6364 (2)	0.0339 (8)	
H32A	−0.2425	0.7797	0.6745	0.051*	
H32B	−0.2395	0.6702	0.6554	0.051*	
H32C	−0.1670	0.7723	0.5604	0.051*	
C51	0.0729 (3)	0.8926 (2)	0.4562 (2)	0.0223 (7)	
H51A	−0.0096	0.8822	0.5099	0.027*	
H51B	0.0788	0.9545	0.3958	0.027*	
C52	0.1645 (3)	0.9111 (3)	0.5029 (2)	0.0373 (5)	
H52A	0.1427	0.9689	0.5314	0.045*	
H52B	0.1593	0.8483	0.5624	0.045*	
C53	0.2931 (3)	0.9363 (3)	0.4241 (2)	0.0373 (5)	
H53A	0.2961	0.9909	0.3591	0.045*	
H53B	0.3204	0.8739	0.4055	0.045*	
C54	0.3782 (3)	0.9728 (2)	0.4664 (2)	0.0373 (5)	
H54A	0.4609	0.9846	0.4145	0.056*	
H54B	0.3736	0.9199	0.5321	0.056*	
H54C	0.3552	1.0375	0.4800	0.056*	
C71	−0.0151 (3)	0.9535 (2)	0.2676 (2)	0.0240 (7)	
H71A	−0.0560	0.9337	0.3450	0.029*	
H71B	−0.0764	0.9442	0.2385	0.029*	
C72	0.0357 (3)	1.0679 (2)	0.2236 (2)	0.0310 (8)	
H72A	−0.0306	1.1104	0.2386	0.047*	
H72B	0.0792	1.0876	0.1473	0.047*	
H72C	0.0911	1.0790	0.2567	0.047*	
C81	0.0710 (3)	0.9022 (2)	0.0649 (2)	0.0228 (7)	
H81A	0.1368	0.9224	−0.0054	0.027*	
H81B	0.0308	0.9644	0.0728	0.027*	
C82	−0.0201 (3)	0.8191 (2)	0.0730 (2)	0.0321 (8)	
H82A	−0.0581	0.8480	0.0219	0.048*	
H82B	−0.0820	0.7955	0.1443	0.048*	
H82C	0.0215	0.7604	0.0581	0.048*	
C101	0.3076 (2)	0.8260 (2)	−0.0339 (2)	0.0203 (6)	
C102	0.3597 (3)	0.9287 (2)	−0.0781 (2)	0.0292 (8)	
H10H	0.3893	0.9589	−0.0389	0.035*	0.600 (2)
C103	0.3687 (3)	0.9873 (2)	−0.1786 (2)	0.0337 (8)	
H10J	0.4077	1.0563	−0.2094	0.040*	0.400 (2)
C104	0.3209 (3)	0.9452 (2)	−0.2337 (2)	0.0314 (8)	
H10A	0.3241	0.9864	−0.3014	0.038*	
C105	0.2682 (3)	0.8433 (2)	−0.1913 (2)	0.0261 (7)	
H10I	0.2362	0.8145	−0.2301	0.031*	0.600 (2)
C106	0.2626 (2)	0.7834 (2)	−0.0915 (2)	0.0198 (6)	
H10K	0.2279	0.7132	−0.0626	0.024*	0.400 (2)
C171	0.5470 (3)	0.3161 (2)	0.4078 (2)	0.0321 (8)	
H17A	0.5767	0.3130	0.3353	0.039*	
H17B	0.5241	0.2442	0.4572	0.039*	
C172	0.6490 (3)	0.3668 (3)	0.4241 (3)	0.0507 (10)	
H17C	0.7179	0.3266	0.4121	0.076*	
H17D	0.6209	0.3687	0.4963	0.076*	
H17E	0.6735	0.4375	0.3743	0.076*	
C181	0.2990 (3)	0.2619 (2)	0.6112 (2)	0.0335 (8)	
H18A	0.3429	0.2026	0.5988	0.040*	
H18B	0.2115	0.2391	0.6389	0.040*	
C182	0.3296 (3)	0.2945 (3)	0.6928 (2)	0.0454 (9)	
H18C	0.3058	0.2363	0.7588	0.068*	
H18D	0.2859	0.3530	0.7055	0.068*	
H18E	0.4166	0.3152	0.6664	0.068*	
N1	0.14074 (19)	0.61957 (17)	0.46025 (16)	0.0173 (5)	
N2	0.20042 (19)	0.75011 (16)	0.26233 (16)	0.0176 (5)	
N4	0.3174 (2)	0.50394 (17)	0.39042 (16)	0.0205 (6)	
Ni1	0.26092 (3)	0.62863 (3)	0.32541 (3)	0.01819 (11)	
N3	0.3797 (2)	0.63970 (18)	0.18985 (16)	0.0218 (6)	
C11	0.3849 (2)	0.7011 (2)	0.0904 (2)	0.0213 (7)	
C14	0.4830 (3)	0.5910 (2)	0.1720 (2)	0.0305 (8)	
C12	0.4970 (3)	0.6906 (2)	0.0100 (2)	0.0318 (8)	
C13	0.5564 (3)	0.6234 (3)	0.0618 (2)	0.0368 (9)	
C131	0.6770 (3)	0.5866 (3)	0.0152 (3)	0.0657 (9)	
H13A	0.6941	0.5962	−0.0598	0.079*	
H13B	0.6727	0.5117	0.0522	0.079*	
C132	0.7794 (3)	0.6439 (3)	0.0232 (3)	0.0657 (9)	
H13C	0.8550	0.6163	−0.0066	0.099*	
H13D	0.7631	0.6346	0.0974	0.099*	
H13E	0.7864	0.7178	−0.0158	0.099*	
C121	0.551 (3)	0.7491 (18)	−0.1075 (8)	0.029 (3)	0.513 (5)
H12A	0.6397	0.7642	−0.1322	0.035*	0.513 (5)
H12B	0.5159	0.8150	−0.1250	0.035*	0.513 (5)
C122	0.5158 (5)	0.6715 (5)	−0.1579 (4)	0.0304 (18)	0.513 (5)
H12C	0.5465	0.7032	−0.2347	0.046*	0.513 (5)
H12D	0.4278	0.6565	−0.1308	0.046*	0.513 (5)
H12E	0.5515	0.6070	−0.1392	0.046*	0.513 (5)
C12B	0.542 (3)	0.734 (2)	−0.1093 (9)	0.029 (3)	0.487 (5)
H12F	0.4750	0.7513	−0.1338	0.035*	0.487 (5)
H12G	0.5868	0.6833	−0.1413	0.035*	0.487 (5)
C12C	0.6292 (7)	0.8338 (6)	−0.1373 (6)	0.065 (3)	0.487 (5)
H12H	0.6599	0.8695	−0.2135	0.097*	0.487 (5)
H12I	0.6970	0.8139	−0.1150	0.097*	0.487 (5)
H12J	0.5846	0.8800	−0.1008	0.097*	0.487 (5)
C108	0.1604 (5)	0.6449 (4)	−0.1065 (4)	0.0352 (8)	0.600 (2)
H10E	0.1277	0.5722	−0.0676	0.053*	0.600 (2)
H10F	0.2222	0.6505	−0.1752	0.053*	0.600 (2)
H10G	0.0952	0.6855	−0.1168	0.053*	0.600 (2)
O2B	0.2130 (3)	0.6839 (2)	−0.0478 (2)	0.0231 (8)	0.600 (2)
O1	0.4351 (3)	1.0869 (2)	−0.2248 (2)	0.0353 (10)	0.600 (2)
C111	0.4968 (5)	1.1267 (4)	−0.1724 (4)	0.0352 (8)	0.600 (2)
H11A	0.5435	1.1935	−0.2199	0.053*	0.600 (2)
H11B	0.5513	1.0778	−0.1528	0.053*	0.600 (2)
H11C	0.4376	1.1362	−0.1090	0.053*	0.600 (2)
C109	0.1601 (7)	0.6923 (6)	−0.2017 (6)	0.0352 (8)	0.400 (2)
H10B	0.1291	0.6752	−0.2500	0.053*	0.400 (2)
H10C	0.0926	0.6892	−0.1369	0.053*	0.400 (2)
H10D	0.2151	0.6426	−0.1848	0.053*	0.400 (2)
O1B	0.2227 (4)	0.7934 (4)	−0.2501 (4)	0.0299 (13)	0.400 (2)
O2	0.3879 (5)	0.9755 (4)	−0.0216 (4)	0.0324 (14)	0.400 (2)
C112	0.4089 (7)	1.0859 (5)	−0.0569 (6)	0.0352 (8)	0.400 (2)
H11G	0.4194	1.1085	−0.0019	0.053*	0.400 (2)
H11D	0.3397	1.1152	−0.0721	0.053*	0.400 (2)
H11E	0.4818	1.1096	−0.1210	0.053*	0.400 (2)

### Atomic displacement parameters (Å^2^)

**Table:**

	*U*^11^	*U*^22^	*U*^33^	*U*^12^	*U*^13^	*U*^23^
C1	0.0172 (16)	0.0229 (17)	0.0141 (15)	0.0023 (13)	−0.0090 (13)	−0.0052 (13)
C2	0.0155 (16)	0.0262 (18)	0.0191 (16)	−0.0013 (13)	−0.0095 (13)	−0.0069 (14)
C3	0.0161 (16)	0.0264 (18)	0.0173 (15)	0.0018 (13)	−0.0084 (13)	−0.0105 (14)
C4	0.0155 (16)	0.0247 (17)	0.0200 (15)	0.0037 (13)	−0.0100 (13)	−0.0115 (14)
C5	0.0159 (16)	0.0232 (17)	0.0216 (16)	0.0084 (13)	−0.0123 (13)	−0.0108 (14)
C6	0.0176 (16)	0.0180 (16)	0.0194 (15)	0.0026 (13)	−0.0087 (13)	−0.0077 (13)
C7	0.0230 (17)	0.0176 (16)	0.0213 (16)	0.0026 (13)	−0.0105 (13)	−0.0053 (13)
C8	0.0234 (17)	0.0180 (16)	0.0197 (16)	0.0020 (13)	−0.0099 (13)	−0.0048 (13)
C9	0.0210 (17)	0.0187 (16)	0.0163 (15)	0.0003 (13)	−0.0099 (13)	−0.0051 (13)
C10	0.0191 (16)	0.0228 (17)	0.0155 (15)	−0.0010 (13)	−0.0066 (13)	−0.0064 (13)
C15	0.0190 (18)	0.046 (2)	0.0280 (18)	0.0165 (16)	−0.0056 (15)	−0.0084 (16)
C16	0.0197 (17)	0.0296 (18)	0.0250 (17)	0.0087 (14)	−0.0092 (14)	−0.0104 (15)
C17	0.0259 (19)	0.0285 (18)	0.0262 (17)	0.0118 (15)	−0.0145 (15)	−0.0137 (15)
C18	0.0321 (19)	0.0220 (17)	0.0227 (16)	0.0084 (14)	−0.0154 (15)	−0.0097 (14)
C19	0.0205 (17)	0.0218 (17)	0.0201 (16)	0.0043 (13)	−0.0097 (13)	−0.0080 (14)
C20	0.0223 (17)	0.0208 (17)	0.0201 (16)	−0.0007 (13)	−0.0099 (14)	−0.0038 (14)
C21	0.0243 (18)	0.0261 (18)	0.0210 (16)	−0.0006 (14)	−0.0042 (14)	−0.0096 (14)
C22	0.057 (2)	0.036 (2)	0.0231 (17)	−0.0043 (17)	−0.0159 (17)	−0.0035 (16)
C31	0.0260 (18)	0.0300 (18)	0.0172 (15)	0.0028 (14)	−0.0050 (13)	−0.0115 (14)
C32	0.0216 (19)	0.045 (2)	0.0378 (19)	0.0098 (16)	−0.0050 (15)	−0.0247 (17)
C51	0.0273 (18)	0.0233 (17)	0.0201 (15)	0.0093 (14)	−0.0107 (14)	−0.0100 (14)
C52	0.0310 (12)	0.0517 (13)	0.0342 (11)	−0.0003 (10)	−0.0105 (9)	−0.0227 (10)
C53	0.0310 (12)	0.0517 (13)	0.0342 (11)	−0.0003 (10)	−0.0105 (9)	−0.0227 (10)
C54	0.0310 (12)	0.0517 (13)	0.0342 (11)	−0.0003 (10)	−0.0105 (9)	−0.0227 (10)
C71	0.0298 (18)	0.0263 (18)	0.0229 (16)	0.0108 (14)	−0.0158 (14)	−0.0106 (14)
C72	0.043 (2)	0.0262 (18)	0.0310 (18)	0.0123 (16)	−0.0204 (16)	−0.0114 (15)
C81	0.0293 (18)	0.0226 (17)	0.0199 (16)	0.0084 (14)	−0.0125 (14)	−0.0081 (13)
C82	0.035 (2)	0.036 (2)	0.0286 (18)	0.0038 (16)	−0.0209 (16)	−0.0049 (15)
C101	0.0195 (17)	0.0245 (17)	0.0138 (14)	0.0021 (13)	−0.0037 (13)	−0.0051 (13)
C102	0.035 (2)	0.0260 (19)	0.0220 (17)	−0.0019 (15)	−0.0057 (15)	−0.0072 (15)
C103	0.043 (2)	0.0188 (18)	0.0245 (18)	−0.0005 (16)	0.0007 (16)	−0.0031 (15)
C104	0.038 (2)	0.030 (2)	0.0154 (16)	0.0108 (16)	−0.0044 (15)	0.0005 (15)
C105	0.0246 (18)	0.036 (2)	0.0190 (16)	0.0078 (15)	−0.0069 (14)	−0.0128 (15)
C106	0.0181 (16)	0.0182 (17)	0.0183 (15)	0.0032 (13)	−0.0034 (13)	−0.0037 (14)
C171	0.0308 (19)	0.036 (2)	0.0354 (19)	0.0161 (16)	−0.0185 (16)	−0.0126 (16)
C172	0.035 (2)	0.062 (3)	0.074 (3)	0.0237 (19)	−0.035 (2)	−0.030 (2)
C181	0.034 (2)	0.0292 (19)	0.0323 (18)	0.0120 (16)	−0.0120 (16)	−0.0041 (16)
C182	0.048 (2)	0.055 (2)	0.0253 (18)	0.0047 (19)	−0.0176 (17)	0.0016 (17)
N1	0.0172 (13)	0.0219 (14)	0.0162 (13)	0.0057 (11)	−0.0086 (11)	−0.0079 (11)
N2	0.0180 (14)	0.0208 (14)	0.0157 (12)	0.0034 (11)	−0.0078 (11)	−0.0065 (11)
N4	0.0182 (14)	0.0279 (15)	0.0177 (13)	0.0083 (12)	−0.0080 (11)	−0.0094 (12)
Ni1	0.0172 (2)	0.0236 (2)	0.0153 (2)	0.00671 (16)	−0.00691 (16)	−0.00747 (17)
N3	0.0179 (14)	0.0298 (15)	0.0152 (13)	0.0077 (11)	−0.0043 (11)	−0.0064 (12)
C11	0.0201 (17)	0.0271 (18)	0.0149 (15)	0.0027 (14)	−0.0055 (13)	−0.0057 (14)
C14	0.0197 (18)	0.044 (2)	0.0232 (17)	0.0130 (16)	−0.0057 (14)	−0.0073 (16)
C12	0.0248 (19)	0.045 (2)	0.0199 (17)	0.0051 (16)	−0.0029 (14)	−0.0090 (16)
C13	0.0248 (19)	0.050 (2)	0.0251 (18)	0.0155 (17)	−0.0024 (15)	−0.0069 (17)
C131	0.0299 (16)	0.114 (3)	0.0406 (15)	0.0344 (17)	−0.0055 (14)	−0.0182 (16)
C132	0.0299 (16)	0.114 (3)	0.0406 (15)	0.0344 (17)	−0.0055 (14)	−0.0182 (16)
C121	0.023 (3)	0.039 (6)	0.0205 (17)	0.003 (4)	−0.0033 (16)	−0.011 (2)
C122	0.023 (4)	0.055 (4)	0.023 (3)	0.004 (3)	−0.009 (3)	−0.025 (3)
C12B	0.023 (3)	0.039 (6)	0.0205 (17)	0.003 (4)	−0.0033 (16)	−0.011 (2)
C12C	0.033 (5)	0.076 (7)	0.047 (5)	−0.006 (5)	0.005 (4)	0.008 (5)
C108	0.044 (2)	0.0275 (19)	0.0384 (19)	−0.0049 (16)	−0.0212 (17)	−0.0089 (16)
O2B	0.026 (2)	0.021 (2)	0.0210 (18)	−0.0026 (15)	−0.0096 (15)	−0.0052 (16)
O1	0.046 (2)	0.023 (2)	0.027 (2)	−0.0070 (18)	−0.0141 (18)	0.0054 (17)
C111	0.044 (2)	0.0275 (19)	0.0384 (19)	−0.0049 (16)	−0.0212 (17)	−0.0089 (16)
C109	0.044 (2)	0.0275 (19)	0.0384 (19)	−0.0049 (16)	−0.0212 (17)	−0.0089 (16)
O1B	0.034 (3)	0.035 (3)	0.026 (3)	0.008 (3)	−0.014 (2)	−0.013 (3)
O2	0.048 (4)	0.021 (3)	0.034 (3)	−0.004 (3)	−0.025 (3)	−0.007 (3)
C112	0.044 (2)	0.0275 (19)	0.0384 (19)	−0.0049 (16)	−0.0212 (17)	−0.0089 (16)

### Geometric parameters (Å, º)

**Table:**

C1—N1	1.371 (3)	C102—O2	1.305 (5)
C1—C20	1.376 (3)	C102—C103	1.386 (4)
C1—C2	1.441 (4)	C102—H10H	0.9500
C2—C3	1.353 (4)	C103—C104	1.377 (4)
C2—C21	1.508 (4)	C103—O1	1.416 (4)
C3—C4	1.462 (3)	C103—H10J	0.9500
C3—C31	1.510 (3)	C104—C105	1.386 (4)
C4—N1	1.387 (3)	C104—H10A	0.9500
C4—C5	1.392 (3)	C105—C106	1.395 (4)
C5—C6	1.403 (3)	C105—O1B	1.472 (5)
C5—C51	1.514 (3)	C105—H10I	0.9500
C6—N2	1.374 (3)	C106—O2B	1.347 (4)
C6—C7	1.450 (3)	C106—H10K	0.9500
C7—C8	1.363 (3)	C171—C172	1.528 (4)
C7—C71	1.509 (4)	C171—H17A	0.9900
C8—C9	1.450 (4)	C171—H17B	0.9900
C8—C81	1.510 (3)	C172—H17C	0.9800
C9—C10	1.394 (3)	C172—H17D	0.9800
C9—N2	1.398 (3)	C172—H17E	0.9800
C10—C11	1.387 (4)	C181—C182	1.531 (4)
C10—C101	1.506 (3)	C181—H18A	0.9900
C15—C16	1.374 (4)	C181—H18B	0.9900
C15—C14	1.378 (4)	C182—H18C	0.9800
C15—H15A	0.9500	C182—H18D	0.9800
C16—N4	1.376 (3)	C182—H18E	0.9800
C16—C17	1.452 (4)	N1—Ni1	1.925 (2)
C17—C18	1.348 (4)	N2—Ni1	1.904 (2)
C17—C171	1.501 (4)	N4—Ni1	1.919 (2)
C18—C19	1.452 (4)	Ni1—N3	1.919 (2)
C18—C181	1.502 (4)	N3—C14	1.378 (3)
C19—N4	1.375 (3)	N3—C11	1.396 (3)
C19—C20	1.375 (4)	C11—C12	1.465 (4)
C20—H20A	0.9500	C14—C13	1.434 (4)
C21—C22	1.528 (4)	C12—C13	1.360 (4)
C21—H21A	0.9900	C12—C12B	1.518 (11)
C21—H21B	0.9900	C12—C121	1.519 (11)
C22—H22A	0.9800	C13—C131	1.511 (4)
C22—H22B	0.9800	C131—C132	1.515 (5)
C22—H22C	0.9800	C131—H13A	0.9900
C31—C32	1.533 (4)	C131—H13B	0.9900
C31—H31A	0.9900	C132—H13C	0.9800
C31—H31B	0.9900	C132—H13D	0.9800
C32—H32A	0.9800	C132—H13E	0.9800
C32—H32B	0.9800	C121—C122	1.59 (2)
C32—H32C	0.9800	C121—H12A	0.9900
C51—C52	1.532 (4)	C121—H12B	0.9900
C51—H51A	0.9900	C122—H12C	0.9800
C51—H51B	0.9900	C122—H12D	0.9800
C52—C53	1.513 (4)	C122—H12E	0.9800
C52—H52A	0.9900	C12B—C12C	1.58 (3)
C52—H52B	0.9900	C12B—H12F	0.9900
C53—C54	1.513 (4)	C12B—H12G	0.9900
C53—H53A	0.9900	C12C—H12H	0.9800
C53—H53B	0.9900	C12C—H12I	0.9800
C54—H54A	0.9800	C12C—H12J	0.9800
C54—H54B	0.9800	C108—O2B	1.434 (5)
C54—H54C	0.9800	C108—H10E	0.9800
C71—C72	1.531 (4)	C108—H10F	0.9800
C71—H71A	0.9900	C108—H10G	0.9800
C71—H71B	0.9900	O1—C111	1.436 (5)
C72—H72A	0.9800	C111—H11A	0.9800
C72—H72B	0.9800	C111—H11B	0.9800
C72—H72C	0.9800	C111—H11C	0.9800
C81—C82	1.528 (4)	C109—O1B	1.416 (8)
C81—H81A	0.9900	C109—H10B	0.9800
C81—H81B	0.9900	C109—H10C	0.9800
C82—H82A	0.9800	C109—H10D	0.9800
C82—H82B	0.9800	O2—C112	1.428 (8)
C82—H82C	0.9800	C112—H11G	0.9800
C101—C106	1.391 (4)	C112—H11D	0.9800
C101—C102	1.394 (4)	C112—H11E	0.9800
			
N1—C1—C20	124.9 (2)	C103—C104—C105	120.7 (3)
N1—C1—C2	110.2 (2)	C103—C104—H10A	119.7
C20—C1—C2	124.0 (2)	C105—C104—H10A	119.7
C3—C2—C1	107.6 (2)	C104—C105—C106	119.5 (3)
C3—C2—C21	127.9 (2)	C104—C105—O1B	122.4 (3)
C1—C2—C21	124.5 (2)	C106—C105—O1B	118.0 (3)
C2—C3—C4	106.4 (2)	C104—C105—H10I	120.2
C2—C3—C31	124.5 (2)	C106—C105—H10I	120.2
C4—C3—C31	128.4 (2)	O2B—C106—C101	118.3 (3)
N1—C4—C5	123.2 (2)	O2B—C106—C105	121.4 (3)
N1—C4—C3	109.5 (2)	C101—C106—C105	120.3 (3)
C5—C4—C3	126.8 (2)	C101—C106—H10K	119.8
C4—C5—C6	119.2 (2)	C105—C106—H10K	119.8
C4—C5—C51	121.2 (2)	C17—C171—C172	113.2 (3)
C6—C5—C51	119.5 (2)	C17—C171—H17A	108.9
N2—C6—C5	124.7 (2)	C172—C171—H17A	108.9
N2—C6—C7	110.1 (2)	C17—C171—H17B	108.9
C5—C6—C7	124.7 (2)	C172—C171—H17B	108.9
C8—C7—C6	107.0 (2)	H17A—C171—H17B	107.7
C8—C7—C71	124.1 (2)	C171—C172—H17C	109.5
C6—C7—C71	128.3 (2)	C171—C172—H17D	109.5
C7—C8—C9	107.1 (2)	H17C—C172—H17D	109.5
C7—C8—C81	122.6 (2)	C171—C172—H17E	109.5
C9—C8—C81	129.5 (2)	H17C—C172—H17E	109.5
C10—C9—N2	122.9 (2)	H17D—C172—H17E	109.5
C10—C9—C8	127.1 (2)	C18—C181—C182	111.5 (2)
N2—C9—C8	109.0 (2)	C18—C181—H18A	109.3
C11—C10—C9	123.5 (2)	C182—C181—H18A	109.3
C11—C10—C101	119.4 (2)	C18—C181—H18B	109.3
C9—C10—C101	116.5 (2)	C182—C181—H18B	109.3
C16—C15—C14	124.8 (3)	H18A—C181—H18B	108.0
C16—C15—H15A	117.6	C181—C182—H18C	109.5
C14—C15—H15A	117.6	C181—C182—H18D	109.5
C15—C16—N4	122.9 (3)	H18C—C182—H18D	109.5
C15—C16—C17	126.0 (3)	C181—C182—H18E	109.5
N4—C16—C17	110.9 (2)	H18C—C182—H18E	109.5
C18—C17—C16	106.4 (2)	H18D—C182—H18E	109.5
C18—C17—C171	128.9 (3)	C1—N1—C4	106.1 (2)
C16—C17—C171	124.7 (3)	C1—N1—Ni1	126.70 (17)
C17—C18—C19	107.1 (2)	C4—N1—Ni1	127.03 (18)
C17—C18—C181	128.3 (3)	C6—N2—C9	106.1 (2)
C19—C18—C181	124.3 (3)	C6—N2—Ni1	127.01 (17)
N4—C19—C20	124.3 (2)	C9—N2—Ni1	126.42 (17)
N4—C19—C18	110.5 (2)	C19—N4—C16	105.1 (2)
C20—C19—C18	124.8 (3)	C19—N4—Ni1	127.50 (18)
C19—C20—C1	122.6 (3)	C16—N4—Ni1	127.42 (19)
C19—C20—H20A	118.7	N2—Ni1—N3	89.77 (9)
C1—C20—H20A	118.7	N2—Ni1—N4	178.32 (10)
C2—C21—C22	112.5 (2)	N3—Ni1—N4	91.08 (9)
C2—C21—H21A	109.1	N2—Ni1—N1	89.26 (9)
C22—C21—H21A	109.1	N3—Ni1—N1	179.03 (10)
C2—C21—H21B	109.1	N4—Ni1—N1	89.89 (9)
C22—C21—H21B	109.1	C14—N3—C11	105.5 (2)
H21A—C21—H21B	107.8	C14—N3—Ni1	125.71 (18)
C21—C22—H22A	109.5	C11—N3—Ni1	128.65 (18)
C21—C22—H22B	109.5	C10—C11—N3	122.7 (2)
H22A—C22—H22B	109.5	C10—C11—C12	127.8 (2)
C21—C22—H22C	109.5	N3—C11—C12	109.4 (2)
H22A—C22—H22C	109.5	N3—C14—C15	124.4 (3)
H22B—C22—H22C	109.5	N3—C14—C13	110.9 (2)
C3—C31—C32	112.1 (2)	C15—C14—C13	124.3 (3)
C3—C31—H31A	109.2	C13—C12—C11	106.6 (2)
C32—C31—H31A	109.2	C13—C12—C12B	122.3 (13)
C3—C31—H31B	109.2	C11—C12—C12B	130.9 (13)
C32—C31—H31B	109.2	C13—C12—C121	123.2 (12)
H31A—C31—H31B	107.9	C11—C12—C121	129.9 (12)
C31—C32—H32A	109.5	C12—C13—C14	107.5 (3)
C31—C32—H32B	109.5	C12—C13—C131	127.8 (3)
H32A—C32—H32B	109.5	C14—C13—C131	124.7 (3)
C31—C32—H32C	109.5	C13—C131—C132	112.7 (3)
H32A—C32—H32C	109.5	C13—C131—H13A	109.1
H32B—C32—H32C	109.5	C132—C131—H13A	109.1
C5—C51—C52	111.5 (2)	C13—C131—H13B	109.1
C5—C51—H51A	109.3	C132—C131—H13B	109.1
C52—C51—H51A	109.3	H13A—C131—H13B	107.8
C5—C51—H51B	109.3	C131—C132—H13C	109.5
C52—C51—H51B	109.3	C131—C132—H13D	109.5
H51A—C51—H51B	108.0	H13C—C132—H13D	109.5
C53—C52—C51	113.4 (2)	C131—C132—H13E	109.5
C53—C52—H52A	108.9	H13C—C132—H13E	109.5
C51—C52—H52A	108.9	H13D—C132—H13E	109.5
C53—C52—H52B	108.9	C12—C121—C122	103.4 (13)
C51—C52—H52B	108.9	C12—C121—H12A	111.1
H52A—C52—H52B	107.7	C122—C121—H12A	111.1
C52—C53—C54	112.2 (2)	C12—C121—H12B	111.1
C52—C53—H53A	109.2	C122—C121—H12B	111.1
C54—C53—H53A	109.2	H12A—C121—H12B	109.0
C52—C53—H53B	109.2	C121—C122—H12C	109.5
C54—C53—H53B	109.2	C121—C122—H12D	109.5
H53A—C53—H53B	107.9	H12C—C122—H12D	109.5
C53—C54—H54A	109.5	C121—C122—H12E	109.5
C53—C54—H54B	109.5	H12C—C122—H12E	109.5
H54A—C54—H54B	109.5	H12D—C122—H12E	109.5
C53—C54—H54C	109.5	C12—C12B—C12C	103.4 (14)
H54A—C54—H54C	109.5	C12—C12B—H12F	111.1
H54B—C54—H54C	109.5	C12C—C12B—H12F	111.1
C7—C71—C72	113.4 (2)	C12—C12B—H12G	111.1
C7—C71—H71A	108.9	C12C—C12B—H12G	111.1
C72—C71—H71A	108.9	H12F—C12B—H12G	109.0
C7—C71—H71B	108.9	C12B—C12C—H12H	109.5
C72—C71—H71B	108.9	C12B—C12C—H12I	109.5
H71A—C71—H71B	107.7	H12H—C12C—H12I	109.5
C71—C72—H72A	109.5	C12B—C12C—H12J	109.5
C71—C72—H72B	109.5	H12H—C12C—H12J	109.5
H72A—C72—H72B	109.5	H12I—C12C—H12J	109.5
C71—C72—H72C	109.5	O2B—C108—H10E	109.5
H72A—C72—H72C	109.5	O2B—C108—H10F	109.5
H72B—C72—H72C	109.5	H10E—C108—H10F	109.5
C8—C81—C82	110.6 (2)	O2B—C108—H10G	109.5
C8—C81—H81A	109.5	H10E—C108—H10G	109.5
C82—C81—H81A	109.5	H10F—C108—H10G	109.5
C8—C81—H81B	109.5	C106—O2B—C108	116.6 (3)
C82—C81—H81B	109.5	C103—O1—C111	122.1 (3)
H81A—C81—H81B	108.1	O1—C111—H11A	109.5
C81—C82—H82A	109.5	O1—C111—H11B	109.5
C81—C82—H82B	109.5	H11A—C111—H11B	109.5
H82A—C82—H82B	109.5	O1—C111—H11C	109.5
C81—C82—H82C	109.5	H11A—C111—H11C	109.5
H82A—C82—H82C	109.5	H11B—C111—H11C	109.5
H82B—C82—H82C	109.5	O1B—C109—H10B	109.5
C106—C101—C102	119.0 (2)	O1B—C109—H10C	109.5
C106—C101—C10	121.4 (2)	H10B—C109—H10C	109.5
C102—C101—C10	119.5 (2)	O1B—C109—H10D	109.5
O2—C102—C103	118.1 (3)	H10B—C109—H10D	109.5
O2—C102—C101	120.7 (3)	H10C—C109—H10D	109.5
C103—C102—C101	120.7 (3)	C109—O1B—C105	121.1 (5)
C103—C102—H10H	119.6	C102—O2—C112	120.0 (5)
C101—C102—H10H	119.6	O2—C112—H11G	109.5
C104—C103—C102	119.7 (3)	O2—C112—H11D	109.5
C104—C103—O1	121.7 (3)	H11G—C112—H11D	109.5
C102—C103—O1	118.5 (3)	O2—C112—H11E	109.5
C104—C103—H10J	120.2	H11G—C112—H11E	109.5
C102—C103—H10J	120.2	H11D—C112—H11E	109.5
			
N1—C1—C2—C3	−5.8 (3)	C103—C104—C105—O1B	−176.3 (3)
C20—C1—C2—C3	163.7 (2)	C102—C101—C106—O2B	179.7 (3)
N1—C1—C2—C21	177.6 (2)	C10—C101—C106—O2B	−2.4 (4)
C20—C1—C2—C21	−12.9 (4)	C102—C101—C106—C105	−0.9 (4)
C1—C2—C3—C4	5.1 (3)	C10—C101—C106—C105	177.0 (2)
C21—C2—C3—C4	−178.5 (2)	C104—C105—C106—O2B	−179.5 (3)
C1—C2—C3—C31	176.3 (2)	C104—C105—C106—C101	1.1 (4)
C21—C2—C3—C31	−7.2 (4)	O1B—C105—C106—C101	178.2 (3)
C2—C3—C4—N1	−2.9 (3)	C18—C17—C171—C172	−101.9 (4)
C31—C3—C4—N1	−173.7 (2)	C16—C17—C171—C172	76.9 (4)
C2—C3—C4—C5	168.7 (3)	C17—C18—C181—C182	91.2 (4)
C31—C3—C4—C5	−2.1 (4)	C19—C18—C181—C182	−82.0 (3)
N1—C4—C5—C6	19.6 (4)	C20—C1—N1—C4	−165.5 (2)
C3—C4—C5—C6	−151.0 (3)	C2—C1—N1—C4	3.8 (3)
N1—C4—C5—C51	−158.2 (2)	C20—C1—N1—Ni1	10.1 (4)
C3—C4—C5—C51	31.2 (4)	C2—C1—N1—Ni1	179.41 (17)
C4—C5—C6—N2	−28.5 (4)	C5—C4—N1—C1	−172.6 (2)
C51—C5—C6—N2	149.4 (3)	C3—C4—N1—C1	−0.6 (3)
C4—C5—C6—C7	142.2 (3)	C5—C4—N1—Ni1	11.8 (4)
C51—C5—C6—C7	−39.9 (4)	C3—C4—N1—Ni1	−176.21 (17)
N2—C6—C7—C8	−0.2 (3)	C5—C6—N2—C9	177.4 (3)
C5—C6—C7—C8	−172.1 (3)	C7—C6—N2—C9	5.5 (3)
N2—C6—C7—C71	171.3 (3)	C5—C6—N2—Ni1	4.7 (4)
C5—C6—C7—C71	−0.5 (5)	C7—C6—N2—Ni1	−167.12 (18)
C6—C7—C8—C9	−5.0 (3)	C10—C9—N2—C6	161.0 (3)
C71—C7—C8—C9	−177.0 (3)	C8—C9—N2—C6	−8.6 (3)
C6—C7—C8—C81	165.7 (2)	C10—C9—N2—Ni1	−26.3 (4)
C71—C7—C8—C81	−6.3 (4)	C8—C9—N2—Ni1	164.09 (18)
C7—C8—C9—C10	−160.4 (3)	C20—C19—N4—C16	174.7 (3)
C81—C8—C9—C10	29.7 (5)	C18—C19—N4—C16	0.8 (3)
C7—C8—C9—N2	8.6 (3)	C20—C19—N4—Ni1	−5.1 (4)
C81—C8—C9—N2	−161.2 (3)	C18—C19—N4—Ni1	−179.01 (17)
N2—C9—C10—C11	3.1 (4)	C15—C16—N4—C19	173.0 (3)
C8—C9—C10—C11	170.8 (3)	C17—C16—N4—C19	−2.1 (3)
N2—C9—C10—C101	−168.2 (2)	C15—C16—N4—Ni1	−7.2 (4)
C8—C9—C10—C101	−0.6 (4)	C17—C16—N4—Ni1	177.76 (18)
C14—C15—C16—N4	−8.4 (5)	C9—C10—C11—N3	10.8 (4)
C14—C15—C16—C17	165.9 (3)	C101—C10—C11—N3	−178.1 (2)
C15—C16—C17—C18	−172.3 (3)	C9—C10—C11—C12	−165.6 (3)
N4—C16—C17—C18	2.6 (3)	C101—C10—C11—C12	5.6 (5)
C15—C16—C17—C171	8.7 (5)	C14—N3—C11—C10	−178.2 (3)
N4—C16—C17—C171	−176.4 (3)	Ni1—N3—C11—C10	−1.5 (4)
C16—C17—C18—C19	−2.0 (3)	C14—N3—C11—C12	−1.3 (3)
C171—C17—C18—C19	177.0 (3)	Ni1—N3—C11—C12	175.4 (2)
C16—C17—C18—C181	−176.1 (3)	C11—N3—C14—C15	−171.7 (3)
C171—C17—C18—C181	2.8 (5)	Ni1—N3—C14—C15	11.4 (4)
C17—C18—C19—N4	0.8 (3)	C11—N3—C14—C13	1.8 (3)
C181—C18—C19—N4	175.3 (2)	Ni1—N3—C14—C13	−175.0 (2)
C17—C18—C19—C20	−173.1 (3)	C16—C15—C14—N3	6.2 (5)
C181—C18—C19—C20	1.4 (4)	C16—C15—C14—C13	−166.5 (3)
N4—C19—C20—C1	−12.1 (4)	C10—C11—C12—C13	177.0 (3)
C18—C19—C20—C1	160.9 (3)	N3—C11—C12—C13	0.3 (4)
N1—C1—C20—C19	9.5 (4)	C10—C11—C12—C12B	−8.4 (14)
C2—C1—C20—C19	−158.4 (3)	N3—C11—C12—C12B	174.9 (14)
C3—C2—C21—C22	−81.0 (3)	C10—C11—C12—C121	3.9 (13)
C1—C2—C21—C22	94.9 (3)	N3—C11—C12—C121	−172.8 (13)
C2—C3—C31—C32	−96.7 (3)	C11—C12—C13—C14	0.8 (4)
C4—C3—C31—C32	72.6 (4)	C12B—C12—C13—C14	−174.3 (12)
C4—C5—C51—C52	62.3 (3)	C121—C12—C13—C14	174.5 (11)
C6—C5—C51—C52	−115.5 (3)	C11—C12—C13—C131	−179.7 (3)
C5—C51—C52—C53	63.6 (3)	C12B—C12—C13—C131	5.2 (13)
C51—C52—C53—C54	169.7 (3)	C121—C12—C13—C131	−6.0 (12)
C8—C7—C71—C72	−82.5 (3)	N3—C14—C13—C12	−1.7 (4)
C6—C7—C71—C72	107.4 (3)	C15—C14—C13—C12	171.9 (3)
C7—C8—C81—C82	−96.7 (3)	N3—C14—C13—C131	178.8 (3)
C9—C8—C81—C82	71.7 (4)	C15—C14—C13—C131	−7.7 (6)
C11—C10—C101—C106	85.9 (3)	C12—C13—C131—C132	99.5 (4)
C9—C10—C101—C106	−102.3 (3)	C14—C13—C131—C132	−81.1 (4)
C11—C10—C101—C102	−96.2 (3)	C13—C12—C121—C122	87.6 (17)
C9—C10—C101—C102	75.6 (3)	C11—C12—C121—C122	−100.3 (15)
C106—C101—C102—O2	170.7 (4)	C13—C12—C12B—C12C	−88.5 (17)
C10—C101—C102—O2	−7.3 (5)	C11—C12—C12B—C12C	97.7 (18)
C106—C101—C102—C103	−1.1 (4)	C101—C106—O2B—C108	175.9 (3)
C10—C101—C102—C103	−179.1 (3)	C105—C106—O2B—C108	−3.5 (5)
O2—C102—C103—C104	−169.1 (4)	C104—C103—O1—C111	−172.8 (4)
C101—C102—C103—C104	2.9 (5)	C102—C103—O1—C111	2.5 (5)
C101—C102—C103—O1	−172.5 (3)	C104—C105—O1B—C109	−174.5 (5)
C102—C103—C104—C105	−2.7 (5)	C106—C105—O1B—C109	8.5 (7)
O1—C103—C104—C105	172.6 (3)	C103—C102—O2—C112	9.6 (7)
C103—C104—C105—C106	0.7 (4)	C101—C102—O2—C112	−162.4 (5)
